# Extracellular Matrix Remodeling in Chronic Liver Disease

**DOI:** 10.1007/s43152-021-00030-3

**Published:** 2021-07-23

**Authors:** Cristina Ortiz, Robert Schierwagen, Liliana Schaefer, Sabine Klein, Xavier Trepat, Jonel Trebicka

**Affiliations:** 1 Translational Hepatology, Department of Internal Medicine I, Goethe University Frankfurt, Theodor-Stern-Kai 7, 60590 Frankfurt, Germany; 2 Institute of General Pharmacology and Toxicology, University Hospital, Goethe University, Frankfurt am Main, Germany; 3 Institute of Bioengineering Catalunya, Barcelona, Spain; 4 Facultat de Medicina, Universitat de Barcelona, 08036 Barcelona, Spain; 5 Centro de Investigación Biomédica en Red en Bioingeniería, Biomateriales y Nanomedicina (CIBER-BBN), 08028 Barcelona, Spain; 6 European Foundation for Study of Chronic Liver Failure, Barcelona, Spain

**Keywords:** Liver fibrosis, Extracellular matrix, Hepatic stellate cell, Collagen, Metalloproteinases, TGF-β1

## Abstract

**Purpose of the Review:**

This review aims to summarize the current knowledge of the extracellular matrix remodeling during hepatic fibrosis. We discuss the diverse interactions of the extracellular matrix with hepatic cells and the surrounding matrix in liver fibrosis, with the focus on the molecular pathways and the mechanisms that regulate extracellular matrix remodeling.

**Recent Findings:**

The extracellular matrix not only provides structure and support for the cells, but also controls cell behavior by providing adhesion signals and by acting as a reservoir of growth factors and cytokines.

**Summary:**

Hepatic fibrosis is characterized by an excessive accumulation of extracellular matrix. During fibrogenesis, the natural remodeling process of the extracellular matrix varies, resulting in the excessive accumulation of its components, mainly collagens. Signals released by the extracellular matrix induce the activation of hepatic stellate cells, which are the major source of extracellular matrix and most abundant myofibroblasts in the liver.

**Graphical abstract:**

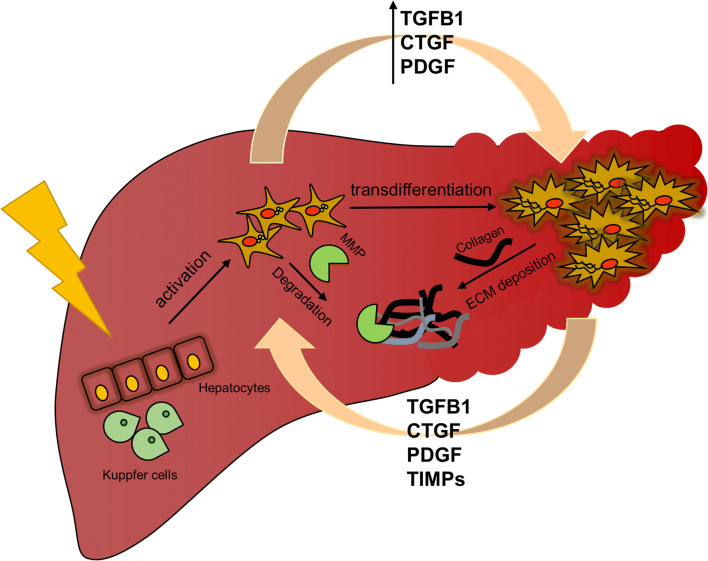

## Introduction

The extracellular matrix (ECM) is a complex cross-linked network of macromolecular proteins that not only provide structural support, but also play an essential role in the development and maintenance of tissue homeostasis [1]. In addition, the interaction between cells and the ECM is bi-directional. Cells are constantly receiving and accepting information from the ECM and, in turn, remodel the ECM in which old proteins are degraded and replaced by new ones in order to maintain the tissue homeostasis [2]. This interaction is mainly mediated by cell surface receptors such as integrins. Integrins are a large family of surface receptors that can signal through the cell membrane in either direction [3]. As a consequence, these integrins transmit signals that regulate cell adhesion, migration, proliferation, apoptosis, survival, or differentiation [4–6]. The ECM proteins contain sites responsible for binding to collagen, the most abundant protein and main structural element of the ECM, as well as for the cross-linking to other ECM proteins such elastin and fibronectin, allowing the degradation by proteases. The common feature of fibrotic diseases is a dysregulation of the ECM composition due to an unbalanced chronic wound-healing process, affecting its structure and biophysical properties [7, 8]. As a consequence, scar formation and tissue fibrosis develop [9].

The liver has a high regenerative potential; however, when the damage becomes persistent, this regeneration turns into chronic diseases, such as fibrosis [10, 11]. Liver fibrosis is usually preceded by inflammation, followed by the activation of the main fibrotic cell type in the liver, the hepatic stellate cells (HSCs) [12]. It has become evident that the ECM proteins represent important mediators of the gain-of-function properties of the HSC during the progression of liver fibrosis [13].

Progressive liver fibrosis can be caused by chronic viral hepatitis, alcohol abuse, non-alcoholic steatohepatitis (NASH), autoimmune or cholestatic disorders, and metabolic diseases [14, 15]. Cirrhosis is the terminal stage of progressive liver fibrosis [16–18]. Hepatic fibrosis is characterized by excess accumulation of ECM [19]. Although initially beneficial, the excessive accumulation of several extracellular proteins leads to an unbalance in the wound-healing process, causing fibrosis [20]. It is important to notice that liver fibrosis is not a unidirectional process, which ultimately will lead to organ failure, but is in principle reversible [21]. In order to monitor the progression of liver fibrosis, there is a need to understand the cellular and molecular mechanisms that shift the balance from healthy to fibrotic liver and, therefore, to develop new antifibrotic therapies in the near future. In this review, we discuss the role of the ECM during liver fibrosis, including the main hepatic cell types and molecular pathways involved in this process, as well as the enzymes and ECM components that contribute and regulate the remodeling and the physical properties of ECM.

## Cell Types Involved in Liver Fibrosis

**Figure 1:**
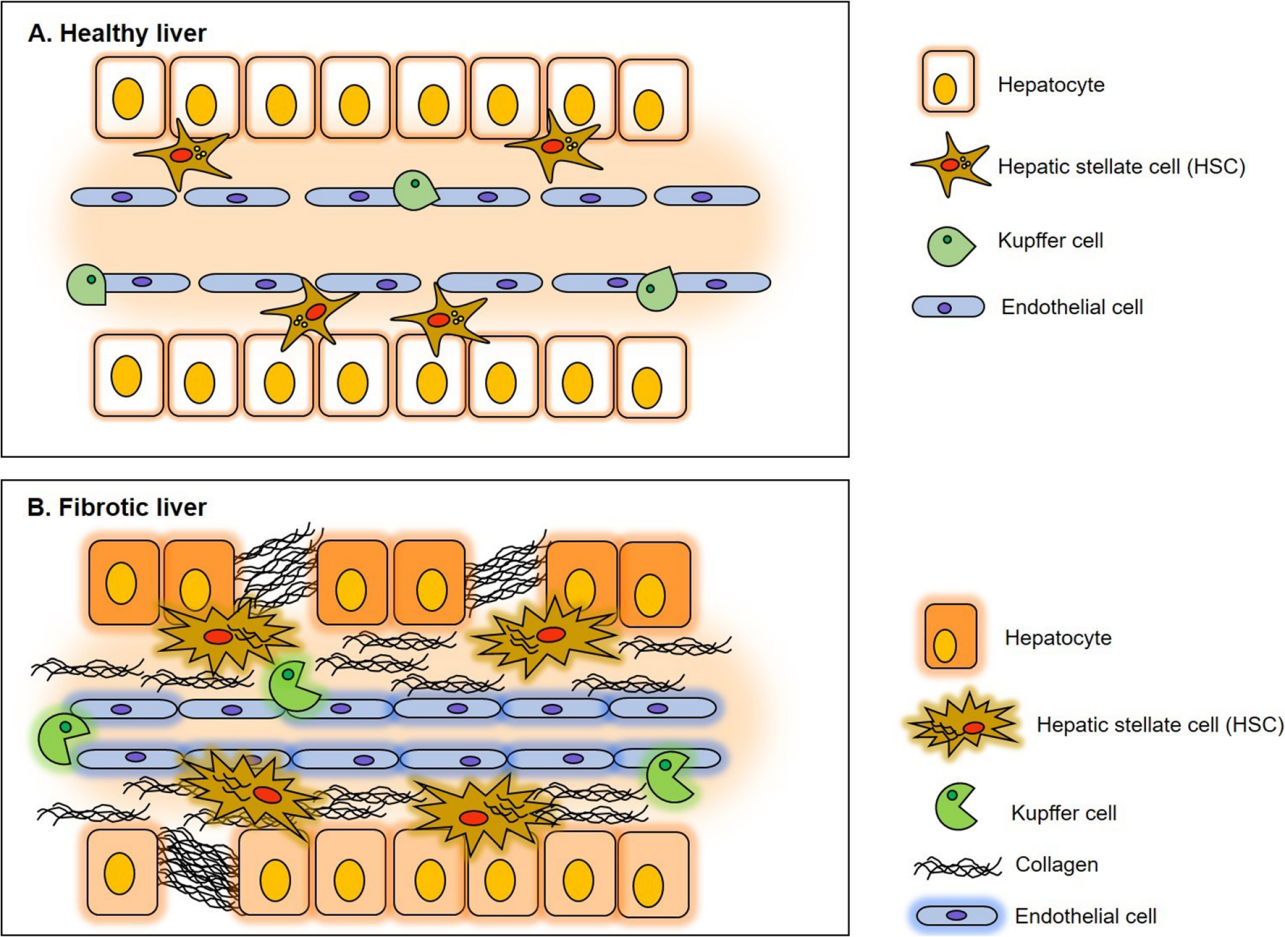
Cellular alterations in liver fibrosis. The main hepatic cell types in the liver are the hepatocytes, the hepatic stellate cells (HSCs), Kupffer cells, and the fenestrated endothelial cells. **a** In a healthy liver, the space between the hepatocytes and endothelial cells is known as the space of Disse, which are located the HSCs. **b** Upon injury, the HSCs become activated and secrete a large amount of extracellular matrix (ECM), which results in gradual thickening of the space. The large amount of ECM, mostly collagen, produced by the activated HSCs leads to the loss of hepatocyte and endothelial fenestrations causing an increase in the portal pressure. The imbalance caused by ECM production and the gain-function of the HSCs cause liver fibrosis.

Understanding liver fibrosis implies knowing how the ECM proteins change during this process, as well as how the cellular players interact with each other. The main cell types in the liver are hepatocytes, Kupffer cells, HSC, liver sinusoidal endothelial cells (LSECs), and cholangiocytes (Figure 1).

Hepatocytes are the major parenchymal cell type in the liver and account for 80–90% of the cells in the liver. As such, they perform the majority of liver functions, including nutrient metabolism and detoxification [22, 23]. Cholangiocytes are a small epithelial cell population (3–5%) that lines up the bile duct system [24]. Their role involves the secretion and absorption of water, electrolytes, and organic solutes [25, 26]. Kupffer cells are the resident macrophages of the liver and largest resident macrophage population in the body (80–90%) [27, 28]. They have a high endocytic and phagocytic capacity, and they play a crucial homeostatic role in the hepatic immune system [29]. LSECs are fenestrated and form a permeable barrier between the blood and the hepatocytes and HSC which facilitates the passage of molecules from the sinusoidal endothelium to the liver parenchyma and contributes to the maintenance of the cellular and hemodynamic homeostasis [30]. HSCs are resident liver cells located in the so-called space of Disse between LSECs and hepatocytes [31••]. In the healthy liver, HSCs are present in a quiescent state, are a reservoir of retinoic acid (vitamin A), and represent approximately 10% of the liver cell population [31••, 32]. LSECs have an important role in the maintenance of the quiescent state of HSCs [33, 34]. In a healthy liver, tissue homeostasis is maintained by intracellular communication between HSCs, hepatocytes, cholangiocytes, Kupffer cells, and LSECs, mainly via cytokines/chemokines. This mechanism is critical for maintenance of the distinct functions of the liver resident cells [35].

Liver injury disturbs tissue homeostasis and causes cell damage in hepatocytes, LSECs, and cholangiocytes leading to necrosis and the release of inflammatory markers, growth factors, reactive oxygen species, and cytokines [17, 29, 36]. Thus, tissue inflammation promotes the attraction and activation of Kupffer cells and HSCs. Although hepatocytes, Kupffer cells, LSECs, and cholangiocytes can contribute to the fibrogenic processes, the primary drivers of fibrosis are the HSCs. Activated HSCs acquire proliferative, migratory, and contractile properties and contribute to 90% of ECM while they develop a myofibroblast-like phenotype and release inflammatory molecules [37].

Portal fibroblasts are another non-parenchymal hepatic cell population and are similar to hepatic stellate cells in some characteristics, since they are also activated in chronic liver injury and contribute to ECM production in liver fibrosis. However, there are also differences between these two cell types. Portal fibroblasts play an important role in cholangitis, since they are located close to the bile ducts [38]. Hepatic stellate cells and portal fibroblasts can be differentiated by their expression profiles. While hepatic stellate cells highly express desmin and scarcely elastin, portal fibroblasts show the opposite expression pattern [39]. The differences in cell marker expression may be used for the research of portal fibroblasts, which play an inferior role in current research efforts when compared to hepatic stellate cells [38].

## ECM Components Implicated in Remodeling

The ECM consists of multiple proteins, such as collagens, elastins, fibronectins, and laminins, that control the cellular phenotype and function [40]. During the transition from healthy to fibrotic liver, the homeostasis between all the ECM proteins and their specific interacting partners is shifted to cause cell inflammation and, finally, contribute to progression of liver fibrosis which in turn is associated with high morbidity and mortality [41].

**Figure 2:**
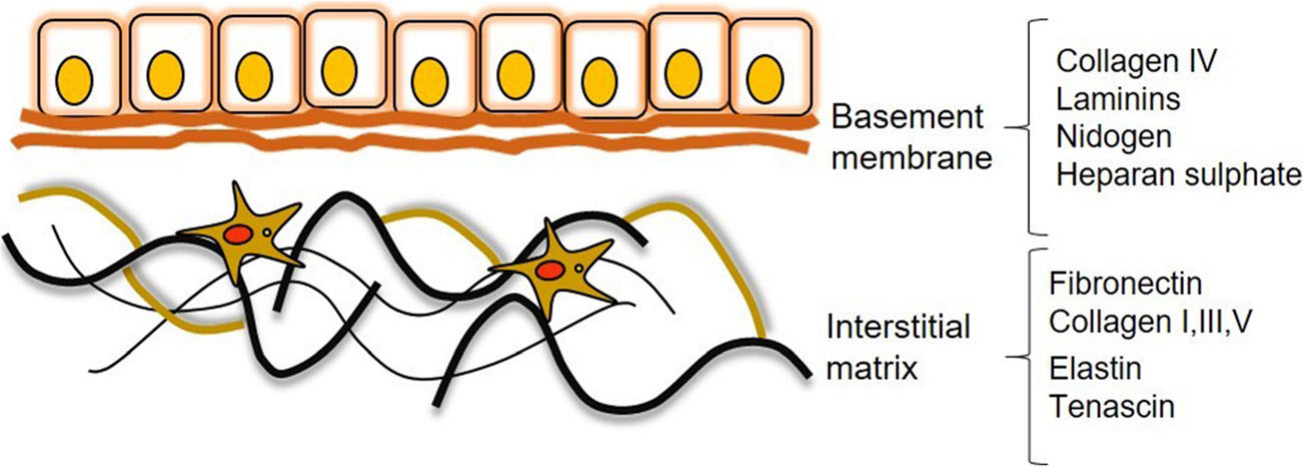
Schematic representation of the two types of extracellular matrix (ECM). The ECM is divided into the loose basement membrane and a compact interstitial matrix. The basement membrane consists in collagen IV, laminins, nicoden, and heparin sulfate proteoglycans. The interstitial matrix is more compact and contain the fibrillary collagens I, III, and V, elastin, fibronectin and tenascin.

There are two types of ECM [40]. The first is the basement membrane (BM) which separates the epithelium from the mesenchyme and the interstitial matrix (IM), produced by fibroblasts and surrounds cells, making up the bulk of the ECM in the body (Figure 2). Laminins, nidogen/entactin, heparan sulfate proteoglycans, and the non-fibrillar collagens, like collagen type IV, are the most abundant components of the BM [42]. In this regard, BM is a highly specialized type of ECM that serves as a reservoir of growth factors that direct cellular functions, provide cell adhesion, and control cell organization and differentiation [40, 43]. Types I, III, and V are the most abundant fibrillary collagens that form the IM, together with elastin, fibronectin, and tenascin [41, 44•]. Type I collagen is the most abundant collagen that is mainly associated with collagen III [45]. Although type V collagen is also found together with collagen I forming heterofibrils, it is not one of the major components of this complex, but it is essential for the structure of tissue [46]. This last characteristic of collagen V makes it unique, since it has been shown that it enhances the stability of collagen fibrils and its gene expression is regulated by TGF-β, ending up in activation of HSCs [47].

The cross-links of the different collagens are mainly involved in the progression of liver fibrosis in the BM and the IM [48]. Due to this cross-link, an increase of up to tenfold in the collagens I, III, and V is detected [49, 50]. Type IV collagen is also increased during liver fibrogenesis, together with laminin and nidogen [51]. The collagen formation observed during fibrosis favors myofibroblast activation, and, although myofibroblasts are the main ECM-producers, other hepatic cell types, such hepatocytes and macrophages, are important regulators of hepatic fibrogenesis and direct effectors of fibrosis progression. The secretion of pro-fibrotic cytokines, chemokines, growth factors, or signaling peptides derived from collagens has been identified as important regulators during liver fibrosis [52].

Endostatin is a potent signaling peptide derived from the C terminus of collagen XVIII that is located in the BM. Collagen XVIII is primarily produced by hepatocytes and is associated to advanced liver fibrosis [53]. In order to reverse the fibrotic phenotype, it has been shown that endostatins ameliorate fibrosis by inhibiting HSC activation [54].

Type IV collagen fragments, from the BM, have been found to have important signaling properties [55]. Six different collagen IV chains have been described up to now (α1–α6), arrestin, canstatin, tumstatin, tetrastatin, pentastatin, and hexastatin [44•, 56–60]. The first three fragments have been shown to inhibit angiogenesis in liver disease as well as endothelial cell proliferation via inhibition of MAPK pathways signaling and inducing apoptosis in endothelial cells [61]. The remaining type IV collagen chains are more limited in distribution than the first ones [51]. Tetrastatin and pentastatin possess similar anti-angiogenic activity and show inhibition of endothelial cell migration [59]; hexastatin was found to regulate endothelial cell adhesion, migration, and proliferation [62]. All together, these signaling peptides are attractive candidates for potential liver fibrosis therapy.

Restin, a signaling peptide of type XV collagen, another BM collagen, is highly abundant in the portal ECM of the liver [63, 64] and shows inhibitory effects on endothelial cell migration but not on their proliferation when the purified protein was assayed in vitro with different endothelial cell lines [65].

Type VI collagen is a microfibrillar collagen found between the IM and the BM that can stimulate the proliferation of mesenchymal cells [66, 67]. During liver fibrosis, it has been found that collagen VI is up to tenfold induced in liver fibrosis [68], and its signaling peptide endotrophin plays a crucial role in fibrosis. In vivo studies showed that in CCl_4_-intoxicated mice, endotrophin was upregulated in injured hepatocytes contributing further to their apoptosis. The inflammatory signal released from injured hepatocytes activates HSCs, resulting in further aggravation of liver fibrosis [69, 70].

Lysyl oxidases (LOX) are the one of the family enzymes that modify the ECM [71, 72]. LOX family enzymes catalyze the cross-linking of collagens through oxidative deamination of lysine residues for the maintenance of the tensile strength and structural integrity of the ECM [73]. At least four different LOX-like (LOXL) proteins (LOXL1, LOXL2, LOXL3, and LOXL4) have been described [71]. LOXL proteins are highly controlled during normal tissue development; however, their aberrant expressions have been reported in liver disease [73]. In the liver, HSCs and portal fibroblasts are the main producers of LOX proteins [74]. During liver fibrosis specifically, LOX and LOXL2, which are absent in healthy tissues but strongly induced in liver fibrosis, have been shown to be upregulated, promoting collagen I cross-linking and its stabilization increasing its resistance to proteolytic degradation, maintaining HSC in an activated state [75]. Increased LOX activity has been detected in sera of patients with hepatic diseases [76, 77], suggesting the LOX family of proteins as a potential biomarker for liver fibrosis. Different approaches have been performed in order to inhibit the activity of this enzyme. For instance, inhibition of LOX with b-aminopropionitrile (BAPN), a potent inhibitor of cross-linking enzymes in the LOX family, has been shown to affect collagen cross-linking making the progression of fibrosis more reversible and delaying the effects of CCl4-intoxication [78]. In mouse models of mild liver fibrosis, it has been shown that inhibition of LOXL2 with a specific monoclonal antibody (AB0023) prevents fibrosis [79]. Further analysis using AB0023 showed efficient inhibition of collagen cross-linking, suppression of fibrosis progression [80, 81•].

Transglutaminases (TGs), in addition to LOX, are a well-characterized family of proteins that are able to covalent cross-link several collagen types from the BM and other ECM proteins like fibronectin or nicogen, increasing their resistance to proteolytic degradation, consequently losing tissue functionality [82–84]. TGs are calcium-dependent enzymes that catalyze the cross-linking, transamidation, or deamidation of proteins [85]. TG2 is the most abundant member and the most studied of the nine members of the TG family that is expressed ubiquitously in many types of tissue, cell membrane, nucleus, and extracellular [86]. During liver inflammation and fibrosis, TG2 is tightly associated with soluble integrins and fibronectins in both, covalently and non-covalently manner, promoting the fibronectin deposition into ECM, and forming stable complexes with both fibronectin and integrins [87]. This association stabilizes the integrin-ECM-fibronectin interaction, making the ECM polymers resistant to proteolytic degradation and therefore, contributing to the progression of fibrosis [88, 89]. In addition, it has been shown that TGF-β enhances the binding of integrin-TG2-fibronectin in fibroblasts and elevates ECM formation during fibrosis [90]. To explore the possibility of TG2 as a target to decrease liver fibrosis, TG2 knockout mice after CCl4-induced liver damage did not show a significant decrease in liver fibrosis when compared to wild-type mice; however, these animals were more susceptible to liver inflammation, which suggest a role of TG2 in control of both inflammation and fibrosis progression [91].

## Molecular Signaling Pathways Involve in Liver Fibrosis

Growth factors, such as transforming growth factor-β (TGF-β), platelet-derived growth factor (PDGF) and connective tissue growth factor (CTGF), as well as oxidative stress are the most potent mediators of inflammatory signals to induce fibrogenesis.

The transforming growth factor-β (TGF-β) signaling is known as the pathway that most hepatic cell types are susceptible to. Three TGF-β isoforms are known in humans, two are fibrogenic, and one seems to have antagonistic properties. While TGF-β1, the major isoform in the liver, is associated with HSC activation and ECM production in liver fibrosis in general and TGF-β2 is associated with biliary liver disease, TGF-β3 might inhibit TGF-β1 and TGF-β2 expression [92–94]. However, data on the role of TGF-β3 in liver fibrosis is scarce.

TGF-β1 signaling is considered one of the main pathways driving HSC activation and the most potent fibrogenic cytokine in the liver [95, 96]. Furthermore, the correlation of TGF-β1 with the severity of liver fibrogenesis demonstrates its importance for liver fibrosis [97]. TGF-β1 is synthesized as a latent precursor with its prodomain and stored in the ECM as part of a large complex [98]. It is activated by mechanical force to induce the conformational changes of the latent complex and release of active TGF-β. Increased contractility by activated HSCs and increased mechanical resistance by higher liver stiffness due to accumulated ECM are the two necessary components to promote TGF-β activation and release [99]. Integrins transmit the force of actin cytoskeleton contractility to the prodomain of the large latent TGF-β complex in the ECM. The linkage of ECM and cytoskeleton via integrins favors the release of TGF-β from the latent TGF-β binding protein complex [100]. Integrins are composed of two subunits, α and β, and each combination has its own binding specificity and signaling properties [95, 101–103]. Although in general the integrins α_v_β3, α_v_β5, α_v_β6, and α_v_β8 can bind this specific sequence of the latent TGF-β1, in liver fibrosis, mainly α_v_β3 and α_v_β6 play a role [104].

While α_v_β6 is barely expressed in normal liver, it is highly expressed in fibrosis [105]. Chemical inhibition of α_v_β6 results in downregulation of pro-fibrotic and upregulation of fibrolytic genes in experimental fibrosis [106]. Several studies have shown in vitro that cells expressing α_v_β_6_ integrin activate TGF-β1, and this interaction can be inhibited by blocking the integrins expressed in the myofibroblasts with antibodies, and thus reducing the fibrotic process [104]. Activated HSCs express α_v_β3, which seems to regulate cell proliferation [107]. Pharmacological inhibition of this integrin resulted in significant collagen reduction mediated by decreased HSC activity [108].

Once activated, TGF-β1 signals by binding to the transmembrane TGF-β type II receptor (TβRII), which recruits and phosphorylates TGF-β type I receptor (TβRI). This process transmits the extracellular TGF-β1 signal towards the intracellular receptor part where the substrates SMAD2 and SMAD3 can bind. SMAD2/3 is activated by phosphorylation and translocates to the nucleus to regulate the transcription of genes maintaining the fibrotic and contractile state of HSCs [109]. TGF-β activation ultimately drives fibrogenesis and ECM production. Specifically, ECM components fibronectin and collagen types I, III, and IV are regulated by TGF-β1 signaling [97, 110]. Increased activation of HSC mediated by TGF-β1 results in proliferation of activated cells and thereby increases the amount of contractile cells which, in turn, promote activation and release of TGF-β1. Therefore, the interaction between HSC and TGF-β1 in fibrogenesis can lead to a vicious cycle of a paracrine activation of HSC. The situation in portal fibroblasts is contrary to HSC. TGF-β1 and TGF-β2 are also produced by portal fibroblasts [111, 112]. While TGF-β1 and TGF-β2 promote HSC proliferation, they inhibit proliferation of portal fibroblast. Overall, this mechanism might provide a growth advantage to HSC over portal fibroblasts [94].

The platelet-derived growth factor (PDGF) signaling pathway functions include regulation of cellular proliferation, cell migration, and stimulation of synthesis of the major components of the ECMs, such as collagen [113]. During liver fibrosis, the PDGF signaling plays an important role in activating HSCs and portal fibroblasts [114]. Among the four secreted cellular PDGF ligands, A-D, PDGF-B, and –D are the most effective in stimulating HSCs and portal myofibroblast proliferation [115]. The biological effects of PDGF are exerted through its ligands, which bind to their receptors, PDGFR-α and PDGFR-β, and therefore inducing proliferation, migration, and cell survival [116]. It has been shown that HSCs express both PDGF receptors; however, only β is upregulated during its activation in vivo and in vitro specifically in liver injury [114]. In contrast to the β isoform, the α isoform expression remained unchanged after liver injury. Given the importance of PDFG signaling in HSC activation during liver fibrosis, much efforts have been taken in order to pharmacologically inhibit the interaction between the ligand and the receptor [117]. There are multikinase inhibitors, sorafenib, which targets tyrosine kinase associated with PDGFR-β, showing downregulation of collagen expression in the livers of fibrotic rats [118, 119].

The connective tissue growth factor (CTGF) is a multi-functional protein which is highly overexpressed during liver fibrosis. CTGF regulates many cellular functions including increased cell proliferation, differentiation, migration, adhesion, and ECM synthesis, and it plays a direct role by interacting with matrix components [120, 121]. CTGF binds integrins, heparin sulfate proteoglycans, and tyrosine kinase receptors that may be located in the matrix or on the cell surface modulating signal transduction into cells [122, 123]. CTGF is a downstream effector of TGF-β [124], and its inhibition has been shown to suppress the TGF-β-dependent induction of ECM proteins, such as collagen and fibronectin [125, 126]. Activated HSCs are the most important source of CTGF, although hepatocytes, portal fibroblast, and cholangiocytes can also contribute to its production [127, 128]. The role of CTGF in liver fibrosis has been studied in vitro using human biopsies from various chronic liver diseases, as well as in activation of HSCs, showing in both cases a strong correlation of CTGF production during liver fibrosis. These results were further confirmed in vivo in CCl_4_-treated and bile duct–ligated (BDL) animals to study liver fibrosis [128]. BDL is an experimental model for cholestatic liver disease which mimics the human primary biliary cirrhosis, and CCl_4_ induces toxic liver fibrosis [129].

Reactive oxygen species (ROS), mediating oxidative stress, are potent pro-fibrotic mediators released mostly by hepatocytes, HSCs and Kupffer cells, that stimulate the production of collagen, acting as a mediator of the fibrogenic action of TGF-β [130, 131]. In liver fibrosis, NADPH oxidases (NOX) are described to be the major sources of ROS [132]. Studies have shown in vivo that NOX1, NOX2, and NOX4 are increased in two models of fibrotic mice, the BDL treatment and the CCl_4_ intoxication. The knockout of NOX1 and NOX4 in these animals resulted in decreased ECM synthesis and overall ROS production. As a consequence, these knockout animals showed a reduction of liver fibrosis, inflammation, and HSC proliferation [133, 134].

The renin-angiotensin system (RAS) is one of the main drivers of HSC activation [135–137, 138•]. RAS is known to be one of the most complex hormonal systems and interplay among its multiple enzymatic peptide and receptor constituents [139]. In the classical RAS, the enzyme renin cleaves its substrate angiotensinogen forming angiotensin I that is in turn, cleaved by angiotensin-converting enzyme (ACE) to produce the angiotensin II (Ang II), the biologically active peptide of the system [139]. In the classical RAS, Ang II activates the Ang II type I receptor (AT1R) to induce, among other biological processes, vasoconstriction. However, an alternative arm of the RAS cleaves Ang II by ACE homologue ACE2 to Angiotensin- [1–7] and stimulates the proto-oncogen *Mas* receptor cascade (MasR) causing vasodilation that generally opposes the actions of Ang II via AT1R [140, 141]. It is well-known that Ang II is the central effector that stimulates HSCs to produce pro-fibrotic cytokines, such as TGF-β1 and CTGF, which further increases hepatic resistance to portal flow and enhanced matrix formation [138•, 142••, 143–145]. However, ACE2 is also the major cellular entry receptor for Severe Acute Respiratory Syndrome Coronavirus 2 (SARS-CoV-2) as a cellular receptor to infect alveolar epithelial cells, causing the severe respiratory disease Coronavirus disease 2019 (COVID-19) in humans [146, 147].

Given the high expression of ACE2 in the liver, specifically in cholangiocytes, resident liver cells located in the bile duct, and at lower levels in hepatocytes, it has been found that a significant number of COVID-19 patients showed abnormal liver test results. Using human organoids as a tool to investigate the SARS-CoV-2 infection, it confirmed that cholangiocytes are the liver cells that express the receptor markers for being infected [148]. Downregulation of ACE2 upon binding of SARS-CoV-2 increases Ang II levels and, consequently, ECM synthesis by activated HSCs. Since SARS viruses are already known to promote pulmonary fibrosis [149], it seems very likely that the same mechanisms can lead to fibrogenesis in the liver too. In this regard, a recently published article supports this hypothesis by analysis in different infected patients and showed that the death of cholangiocytes is induced by SARS-CoV-2. As a consequence, hepatocytes release proinflammatory cytokines that can easily produce liver injury [150, 151].

## Matrix Stiffness and Inflammation

One of the characteristic events as the liver becomes fibrotic is that the ECM stiffness increases by an extensive deposition of their extracellular proteins, including fibrillar and membrane collagens, affecting the cellular behavior [152]. The concept of stiffness has been analyzed years ago [153] and, nowadays, it is well-known that it can contribute to the mechanical properties of the ECM proteins. Matrix stiffness affects the behavior of the HSCs, including growth, motility, adhesion, and differentiation into myofibroblasts [154].

Fibrosis is associated with an increased matrix stiffness as a consequence of excessive collagen deposition and cross-linking [155]. This stiffness is detected by HSC surface receptors, the integrins, allowing the HSC activation [156]. It has been shown that the ECM components are responsible for increased matrix stiffness and, as a consequence, promotes HSC activation via cytoskeleton modulation [157•]. An important aspect concerning matrix stiffness during liver fibrosis progression is the imbalance of the main enzymes implicated in the ECM degradation, such as the matrix metalloproteinases (MMPs) and their inhibitors. This family of proteins includes the tissue inhibitors of metalloproteinases (TIMPs) [158, 159]. Up to now, 25 different MMPs have been identified that regulate the degradation of most ECM proteins [160]. In a normal liver, MMPs are capable to degrade any protein from the ECM in order to maintain tissue homeostasis [161]. However, when HSCs are activated and collagen accumulates excessively, the matrix regeneration fails, leading to an increase in the stiffness [162]. In this regard, in vitro studies using HSCs cultured on different substrates of varying rigidity, that mimics healthy and fibrotic liver tissue, have shown that increasing fibrotic matrix stiffness downregulates MMP-9 gene expression. As a consequence, this increase in rigidity promotes the secretion of TIMP-1 which inhibits permanently the MMP activity to degrade ECM, promoting fibrosis perpetuation [152]. In addition, other in vitro studies using activated HSCs showed that activation of the HSCs is associated as well with increased MMP-2 and MMP-14 protein expression [163].

Inflammation is a crucial mechanism promoting liver fibrosis by initiation of a protective response to tissue injury [164, 165]. The components of the ECM are the main regulators of tissue inflammation [166]. More specifically, the ECM-derived damage-associated molecular patterns (DAMPs) activate Toll-like receptors (TLRs) and the inflammasome in order to induce tissue inflammation [167, 168]. ECM proteoglycans and their fragments, such as biglycan and decorin, are the most prominent and well-characterized ECM-derived DAMPs [169–171]. Both can interact with TLR2 and TLR4 inducing pro- and anti-inflammatory effects and recruiting macrophages [172, 173]. Macrophages sense changes in matrix stiffness through mechanotransduction and respond by regulating the TLR-mediated inflammatory signal. In this context, the release of the proinflammatory cytokine TNF-α, mainly involved in liver inflammation leading to fibrosis, is increased in response to TLR4 and TLR9 stimulation [174].

## Biophysical Mechanisms of ECM Stiffness Sensing

ECM stiffness does not remain unrecognized by adjacent cells. It has been reported that fibroblasts tend to move from softer to stiffer regions [175]. This directed migration is called durotaxis, and the underlying mechanisms are less well-understood than in chemotaxis in which specific cell membrane receptor sense is a gradient in a soluble factor. To detect a gradient in stiffness, cells need to actively apply a pulling force to the substrate. Pulling forces are generated by the actomyosin cytoskeleton, and they can be transmitted to integrins through specific protein complexes called focal adhesions [176]. Focal adhesions contain mechanosensitive proteins such as talin and vinculin. Talin links the cytoskeleton to ECM via intregrins and vinculin shows increased binding to focal adhesion complexes with applied force. The interaction of both proteins is important to sense forces. Under force, the talin structure unfolds to expose multiple binding sites for vinculin. Without an applied force, the talin structure contains less vinculin binding site [177]. The dynamics of the linear physical connection between the actomyosin cytoskeleton and the ECM has been understood through the so-called clutch models [178]. In their simplest form, these models include the deformability of the matrix, the on and off rates of the different integrins and adaptor molecules that connect the actomyosin cytoskeleton and the ECM, and the physical relation that links force and velocity generated by myosin motors [179]. The force generated by the actomyosin cytoskeleton is contractile and results in a flow of actin towards the center of the cell, called retrograde flow. In general, this flow is inversely proportional to the traction force exerted by the cell migrating on the ECM [178].

When actomyosin-generated forces build up at focal adhesions, two main outcomes are possible depending on the rigidity of the substrate [180•]. On soft substrates, force builds up slowly and integrins unbind from the ECM before talin unfolds, resulting in rapid actin retrograde flow. By contrast, on stiff substrates force builds up fast and talin unfolds before integrins unbind, resulting in slow actin flow. Talin unfolding on stiff substrates triggers a reinforcement feedback loop by which vinculin is recruited to focal adhesions. Downstream of vinculin binding to talin, both actin and integrins are recruited to focal adhesions and mediate their reinforcement [180•]. Clutch models thus provide a conceptual framework that explains how rigidity sensing can be tuned through the regulation of vinculin and talin binding rates, activation of mechanochemical switches, and changes in cellular contractility. This mechanism has also been suggested to explain durotaxis, i.e., the ability of single cells and cell collective to migrate from soft to rigid ECM [181].

## Concluding Remarks

In this review, we clearly point out the prime importance of the complex interplay between distinct cells and the ECM for health and disease in the liver, where TGF-β occupies an outstanding position as a key mediator of this interaction. ECM not only plays an important role as the framework of the liver, but it also participates actively in the cellular processes as well as in the cell-cell communication. ECM components and properties modulate physiological and pathological processes in the liver. ECM research remains a dynamic field, and recent findings might help to fight contemporary health threats like COVID-19. The complex interaction of ECM with the surrounding cells and compartments needs to be taken into account when designing diagnostic tools and therapeutical antifibrotic strategies.
